# Establishment of HLA-DR4 Transgenic Mice for the Identification of CD4^+^ T Cell Epitopes of Tumor-Associated Antigens

**DOI:** 10.1371/journal.pone.0084908

**Published:** 2013-12-30

**Authors:** Junji Yatsuda, Atsushi Irie, Kumiko Harada, Yayoi Michibata, Hirotake Tsukamoto, Satoru Senju, Yusuke Tomita, Akira Yuno, Masatoshi Hirayama, Mohammad Abu Sayem, Naoki Takeda, Isao Shibuya, Shinji Sogo, Fumihiro Fujiki, Haruo Sugiyama, Masatoshi Eto, Yasuharu Nishimura

**Affiliations:** 1 Department of Immunogenetics, Graduate School of Medical Sciences, Kumamoto University, Kumamoto, Japan; 2 Department of Urology, Graduate School of Medical Sciences, Kumamoto University, Kumamoto, Japan; 3 Department of Biotechnology and Genetic Engineering, Mawlana Bhashani Science and Technology University, Tangail, Bangladesh; 4 Division of Transgenic Technology, Institute of Resource Development and Analysis, Kumamoto University, Kumamoto, Japan; 5 Microbiological Research Institute, Otsuka Pharmaceutical Co., Ltd, Tokushima, Japan; 6 Department of Cancer Immunology, Osaka University Graduate School of Medicine, Osaka, Japan; 7 Department of Functional Diagnostic Science, Osaka University Graduate School of Medicine, Osaka, Japan; Mie University Graduate School of Medicine, Japan

## Abstract

Reports have shown that activation of tumor-specific CD4^+^ helper T (Th) cells is crucial for effective anti-tumor immunity and identification of Th-cell epitopes is critical for peptide vaccine-based cancer immunotherapy. Although computer algorithms are available to predict peptides with high binding affinity to a specific HLA class II molecule, the ability of those peptides to induce Th-cell responses must be evaluated. We have established HLA-DR4 (HLA-DRA*01:01/HLA-DRB1*04:05) transgenic mice (Tgm), since this HLA-DR allele is most frequent (13.6%) in Japanese population, to evaluate HLA-DR4-restricted Th-cell responses to tumor-associated antigen (TAA)-derived peptides predicted to bind to HLA-DR4. To avoid weak binding between mouse CD4 and HLA-DR4, Tgm were designed to express chimeric HLA-DR4/I-E^d^, where I-E^d^ α1 and β1 domains were replaced with those from HLA-DR4. Th cells isolated from Tgm immunized with adjuvant and HLA-DR4-binding cytomegalovirus-derived peptide proliferated when stimulated with peptide-pulsed HLA-DR4-transduced mouse L cells, indicating chimeric HLA-DR4/I-E^d^ has equivalent antigen presenting capacity to HLA-DR4. Immunization with CDCA1_55-78_ peptide, a computer algorithm-predicted HLA-DR4-binding peptide derived from TAA CDCA1, successfully induced Th-cell responses in Tgm, while immunization of HLA-DR4-binding Wilms' tumor 1 antigen-derived peptide with identical amino acid sequence to mouse ortholog failed. This was overcome by using peptide-pulsed syngeneic bone marrow-derived dendritic cells (BM-DC) followed by immunization with peptide/CFA booster. BM-DC-based immunization of KIF20A_494-517_ peptide from another TAA KIF20A, with an almost identical HLA-binding core amino acid sequence to mouse ortholog, successfully induced Th-cell responses in Tgm. Notably, both CDCA1_55-78_ and KIF20A_494-517_ peptides induced human Th-cell responses in PBMCs from HLA-DR4-positive donors. Finally, an HLA-DR4 binding DEPDC1_191-213_ peptide from a new TAA DEPDC1 overexpressed in bladder cancer induced strong Th-cell responses both in Tgm and in PBMCs from an HLA-DR4-positive donor. Thus, the HLA-DR4 Tgm combined with computer algorithm was useful for preliminary screening of candidate peptides for vaccination.

## Introduction

Tumor cells express various proteins different from those of normal somatic cells, or over-express proteins at higher levels than in normal cells. Thus, peptides derived from these proteins may be specifically recognized by T lymphocytes. Of the two tumor-specific T lymphocyte subsets, CD8^+^ cytotoxic T lymphocytes (CTL) recognize tumor-associated antigen (TAA)-derived peptides in the context of MHC class I molecules (MHC-I), whereas CD4^+^ helper T (Th) cells respond to peptide-MHC class II (MHC-II) complexes. Because of their ability to eradicate malignant cells directly, CTLs have long been defined as the critical effector cells in anti-tumor immunity, although Th cells can also induce robust anti-tumor immune responses [1-3]. It is well accepted that tumor-specific Th cells maintain the anti-tumor responses of CTLs by licensing dendritic cells (DC) to effectively prime CTLs [4,5] by generating effective memory CTL [6,7], or by direct stimulation of effector CTLs [8]. Thus, identification and vaccination of Th-cell epitopes that activate tumor-specific Th cells is might be a promising method to induce effective anti-tumor immunity in tumor-bearing hosts. Although it may be possible to identify such Th-cell epitopes using human peripheral blood mononuclear cells (PBMCs) isolated from healthy donors or cancer patients, it requires frequent bleedings and assays using a large number of overlapping peptides, which is in many cases a very long and labor-intensive procedure. 

Utilizing a computer algorithm for the prediction of peptides with high binding affinity to a specific HLA/MHC molecule might be a convenient alternative. For example, SYFPEITHI (http://www.syfpeithi.de/) and BIMAS (http://www-bimas.cit.nih.gov) are powerful tools to predict MHC class I binding and therefore potential CTL-epitopes. On the other hand, the confidence of predicting peptides that bind to a specific HLA/MHC-II molecule is still under discussion and the ability of those peptides to induce Th-cell responses must be evaluated. Therefore, we established transgenic mice expressing HLA-DR4 (HLA-DRA/HLA-DRB1*04:05). of which allele frequency is 13.6% in Japanese population, to evaluate HLA-DR4-restricted Th-cell responses to tumor-associated antigen (TAA)-derived peptides predicted to bind to HLA-DR4. Using those mice, immunization with peptides known to be recognized by HLA-DR4-restricted human Th cells successfully induced peptide-specific and HLA-DR4-restricted mouse Th cell responses, demonstrating the validity of the HLA-DR4 Tgm for the preliminary screening of novel TAA-derived and HLA-DR4-restricted Th epitopes, which could be clinically applicable for peptide vaccine-based cancer immunotherapy in the future.

## Materials and Methods

### Ethics Statement

This study was approved by the animal research committee of Kumamoto University (Permission Number: B25-115). The mice were maintained at the Center for Animal Resources and Development of Kumamoto University, and were handled in accordance with the animal care guidelines of Kumamoto University. 

The Institutional Review Board of Kumamoto University approved the research protocol for collecting and using PBMCs from healthy donors performed in this study with written informed consent.

### Gene Constructs of HLA-DRA/I-E^d^α and HLA-DRB1*0405/I-E^d^β

**Figure 1:**
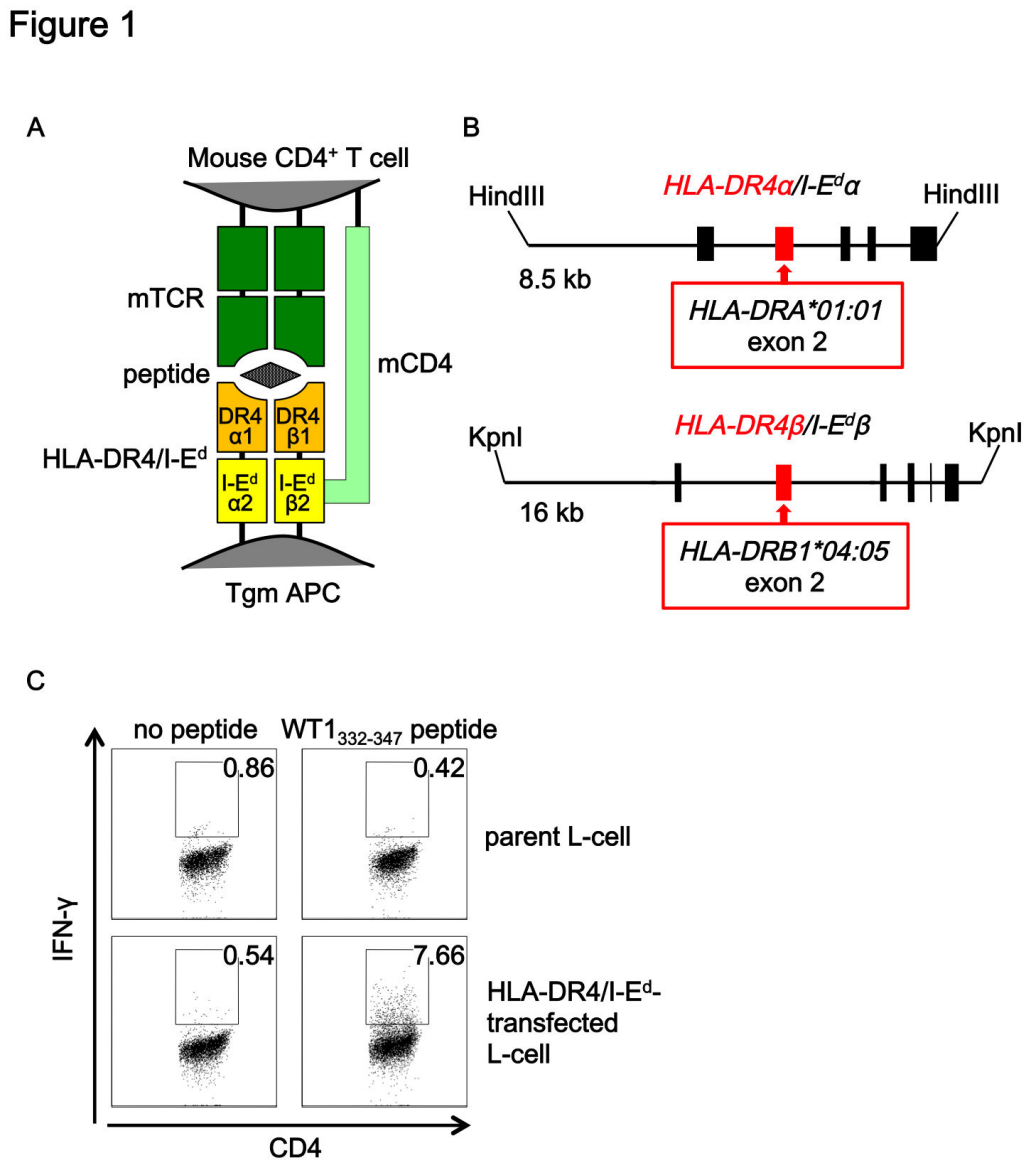
Transgenes encoding chimeric HLA-DR4/I-E^d^ molecule. (A) Schematic diagram of mouse T cell receptor (mTCR, green), mouse CD4 (mCD4, pale green) and HLA-DR4/I-E^d^ chimeric molecules (orange and yellow). (B) To avoid inter-species interactions between mCD4 and HLA-DR4, the second exons of *I-E* ^*d*^ *α* and *I-E* ^*d*^ *β* genes encoding α1 and β1 domains were substituted with those of HLA-DRA and *HLA-DR4B* genes (red boxes), respectively. The transgenes contain the endogenous I-E^d^α and I-E^d^β promoter regions spanning 3.2 kb and 5.2 kb of 5'-untranslated regions, respectively. (C) WT1_332-347_ peptide-pulsed L-cells expressing HLA-DR4/I-E^d^ stimulated IFN-γ production by the WT1_332-347_ peptide-specific and HLA-DR4-restricted human Th clone (gated on CD4).

To avoid possible problems caused by inter-species interactions between mouse CD4 and HLA-DR, a chimeric HLA-DR4/I-E^d^ molecule, of which translated α1 and β1 domains were derived from HLA-DR4 and the other domains were derived from respective I-E^d^ α and β molecules, was constructed [9]. The genomic fragments of exon 2 of *I-E* ^*d*^ *α* and β *genes* were replaced with those of HLA-DRA*01:01 and HLA*DRB1*04:05 genes, respectively. The 8.5 kb HindIII and 16 kb KpnI fragments were used for the chimeric *HLA-DRA/I-E* ^*d*^α and HLA-DRB1*0405/I-E ^*d*^β transgenes, which contain endogenous I-E^d^α and I-E^d^β promoter regions spanning 3.2 kb and 5.2 kb of 5'-untranslated regions, respectively (Figure 1). The *HLA-DR4/I-E* ^*d*^ transgenes were co-injected into C57BL/6 fertilized eggs [10] and were transferred into the oviduct of pseudopregnant ICR mice. A total of 25 pups were obtained and at 4-5 weeks after birth, their PBMCs were subjected to flow cytometric analyses using anti-HLA-DRα-chain monoclonal antibody (mAb) L243 and anti-HLA-DR4β-chain mAb TAL15.1. In addition, genomic PCR analyses was used using the following primer sets: 5'-CACCCAGACACTGTTTCTTC-3' and 5'-CAAAGCTGGCAAATCGTC-3' for *HLA-DRA/I-E* ^*d*^α and 5'-CCCGTTAGTTGTGGTGACCT-3' and 5'-GCACTGTGAAGCTCTCACCA-3' for HLA-DRB1*04:05/I-E ^*d*^β, respectively after preparation of genomic DNA using DNeasy Blood and Tissue Kit (Qiagen, Venlo, Netherlands). The presence and expression of *HLA-DR4/I-E* ^*d*^ transgenes were confirmed by PCR and flow cytometry and positive offspring were crossed with wild-type (WT) C57BL/6 mice. 

### Cell lines

L-DR4, genetically engineered mouse fibroblast L cells that express HLA-DR4 (HLA-DRA*01:01/DRB1*04:05), were maintained in DMEM supplemented with 10% fetal calf serum (FCS) and 50 U/ml penicillin/streptomycin in a 5% CO_2_ atmosphere at 37°C.

### Generation of bone marrow-derived dendritic cells

Bone marrow-derived dendritic cells (BM-DCs) were generated as described by Inaba et al [11]. Brieﬂy, bone marrow cells obtained from mouse thighbones and hind limbs were cultured at 1 × 10^7^ cells per well in 10 ml of RPMI 1640 supplemented with mouse GM-CSF 10 ng/ml, 10% FCS, 50 μM 2-mercaptoethanol (2ME) and 50 U/ml penicillin/streptomycin. On day 14, the cells were recovered for antigen presentation.

### Synthetic peptides

Peptides were synthesized by Biomatik (Cambridge, Ontario, Canada). The peptides used in this study were: CMV-derived CMV-egH_290–302_ peptide (SYLKDSDFLDAAL) [12], WT1-derived WT1_332-347_ peptide (KRYFKLSHLQMHSRKH) [13], CDCA1-derived CDCA1_55-78_ peptide (IVYGIRLEHFYMMPVNSEVMYPHL) [14], KIF20A -derived KIF20A_494-517_ peptide (TLHVAKFSAIASQLVHAPPMQLGF) [15], DEPDC1-derived DEPDC1_191-213_ peptide (RYVILIYLQTILGVPSLEEVINP) and a negative control DEPDC1_60-85_ peptide (NSNFGPEVTRQQTIQLLRKFLKNHVI). The purity of the peptides was routinely >95%. Lyophilized peptides were dissolved in DMSO and stored at −20°C.

### Flow Cytometry

After hemolysis, PBMCs were stained with various PE- or FITC-conjugated mAbs. The cells were analyzed by FACScan (BD Biosciences, Franklin Lakes, NJ, USA) using Cell Quest software. PE-conjugated anti-HLA-DR-α-chain mAb L243 was from BD and FITC-conjugated anti-HLA-DR-β-chain mAb TAL15.1, PE-conjugated anti-I-A^b^ mAb, PE-conjugated anti-mouse MHC-II mAb, FITC-conjugated anti-B220 mAb, PE- or FITC-conjugated anti-mouse CD4 mAb were from eBioscience (San Diego, CA, USA).

### Intracellular IFN-γ staining

Parental L-cells and L cells expressing HLA-DR4/I-E^d^ were incubated with or without 10 μg/ml of WT1_332-347_ peptide for 3 h, washed extensively, and then used as a stimulator. L cells were co-incubated with HLA-DR4-restricted WT1_332-347_ peptide-specific CD4^+^ T cell clones [13] in the presence of 2 μg/ml CD28/CD49d Costimulatory Reagent (BD Biosciences) and 10 μg/ml Brefeldin A (Sigma-Aldrich, St Louis, MO, USA) for 5 h. Intracellular staining of IFN-γ was performed using BD Cytofix/Cytoperm Buffer (BD Biosciences) according to the manufacturer’s procedures after surface staining of CD4 molecules. PE-conjugated anti-IFN-γ mAb and FITC-conjugated anti-CD4 mAb were from BD Biosciences. The cells were analyzed with FACSAria (BD Biosciences). The data were analyzed with FlowJo software.

### Immunization of mice

Mice were primed either in the tail base with 50 μl of peptides in PBS (1 μg/μl) emulsified with 50 μl of Complete Freund’s Adjuvant (CFA, Sigma-Aldrich) or by intravenous injection with peptide-pulsed BM-DCs (5 × 10^5^). Seven days after the first immunization, mice were boosted with 50 μl of peptide in PBS (1 μg/μl) emulsified with 50 μl of Incomplete Freund’s Adjuvant (IFA, Sigma-Aldrich) or CFA. On day 14, the inguinal lymph nodes or spleen cells were collected and cultured with the peptides and IL-2 (20 U/ml) for 7 days. Th cells were isolated by MACS beads (Miltenyi Biotec, Bergish-Gladbach, Germany) according to the manufacturer’s instruction and assayed as described below. In some experiments, the harvested lymph nodes cells on day 14 were subjected to *ex vivo* proliferation assay.

### T cell proliferation assay and blocking experiment

Purified Th cells were co-cultured with peptide-pulsed L-DR4 cells for 48 h in RPMI 1640 medium containing 8% FCS, 50 U/ml penicillin/streptomycin and 2ME, then pulsed with 1 μCi [^3^H-] thymidine and cultured for another 17 h. The cells were harvested and incorporated radioactivity was counted using a scintillation counter (I450 Microbeta, Trilux, PerkinElmer). To confirm the HLA-DR4 restriction, single cell-suspension of inguinal LN cells was cultured with or without peptide in the presence or absence of anti-HLA-DR blocking mAb L243 for 48 h. Then, incorporated radioactivity was counted as described above. 

### IFN-γ ELISPOT assay

The ELISPOT assay was performed as described previously [14]. Briefly, Th cells were incubated in triplicate in ELISPOT plates (BD Biosciences) under the presence of the indicated peptides (10 μg/ml) and L-DR4 as antigen presenting cells. According to the manufacturer's instructions, the plates were incubated for 18 h at 37°C and IFN-γ-positive spots were quantified using Eli photo (Minerva Tech, Tokyo, Japan).

### Generation of antigen-specific human CD4^+^ T cells

We obtained PBMCs from HLA-DR4-positive healthy donors (genotyped by the HLA Laboratory, Kyoto, Japan). Induction of antigen-specific CD4^+^ T cells was performed as follows:. We isolated the PBMCs from the heparinized blood of Japanese healthy donors by means of Ficoll-Conray density gradient centrifugation. CD4^+^ T cells and CD14^+^ cells were purified from PBMCs. Monocyte-derived DCs were generated from CD14^+^ cells and used as antigen-presenting cells (APCs) to induce antigen-specific CD4^+^ T cells. CD14^+^ cells co-cultured with human GM-CSF (100 ng/ml) and human IL-4 (10 ng/ml) in a 10 cm tissue culture dish for 7 days. On day 5, OK-432 (0.1 KE/ml) were added into the dish. On day 7, DCs (1 × 10^4^ /well) were pulsed with 10 µg/ml peptide for 3 h, irradiated (45 Gy), and subsequently mixed with CD4^+^ T cells (3 × 10^4^ /well) in 200 µL AIM-V (Life Technologies) supplemented with 5% human decomplemented plasma in each well of a 96-well, flat-bottomed culture plate. After 7 days, half of the medium was removed from each culture, and fresh medium (100 µl/well) containing irradiated (50 Gy) autologous PBMCs (1 × 10^5^) pulsed with peptide (10 µg/ml) and 5 ng/ml recombinant human IL-7 (rhIL-7) was added. Two days after the second stimulation with peptide, rhIL-2 was added to each well (10 IU/ml). A week later, the stimulated CD4^+^ T cells in each well were analyzed for specificity in IFN-γ ELISPOT assays. The T cells showing a specific response to the cognate peptide were transferred to 24-well plates and re-stimulated at weekly intervals with irradiated autologous PBMCs (1 × 10^6^ /well) pulsed with the peptide in medium supplemented with rhIL-2 (20 IU/ml) and rhIL-7 (5 ng/ml) [14]. 

## Results

### Expression and characterization of the chimeric HLA-DR4/I-E^d^ molecule *in vitro*

The *HLA-DRA/I-E* ^*d*^α and HLA-DRB1*0405/I-E ^*dβ*^ chimeric transgenes were first co-transfected into mouse fibroblast L cells and cell surface expression of HLA-DR4/I-E^d^ molecules were confirmed by positive staining with FITC-conjugated anti-HLA-DR antibody (TAL15.1) by flow cytometry (data not shown). HLA-DR4-restricted and WT1_332-347_ peptide-specific T cell clones [13] produced IFN-γ in an HLA-DR4-restricted and a peptide-specific manner when T cells were co-cultured with the TAL15.1-positive L-cells pulsed with WT1_332-347_ peptide (Figure 1C). These results suggested that the chimeric HLA-DR4/I-E^d^ molecules were expressed and could stimulate HLA-DR4-restricted T-cell responses by transduction of the *HLA-DRA/I-E* ^*d*^α and HLA-DRB1*0405/I-E ^*d*^β chimeric transgenes into the mice.

### Characterization of HLA-DR4 Transgenic Mice

**Figure 2:**
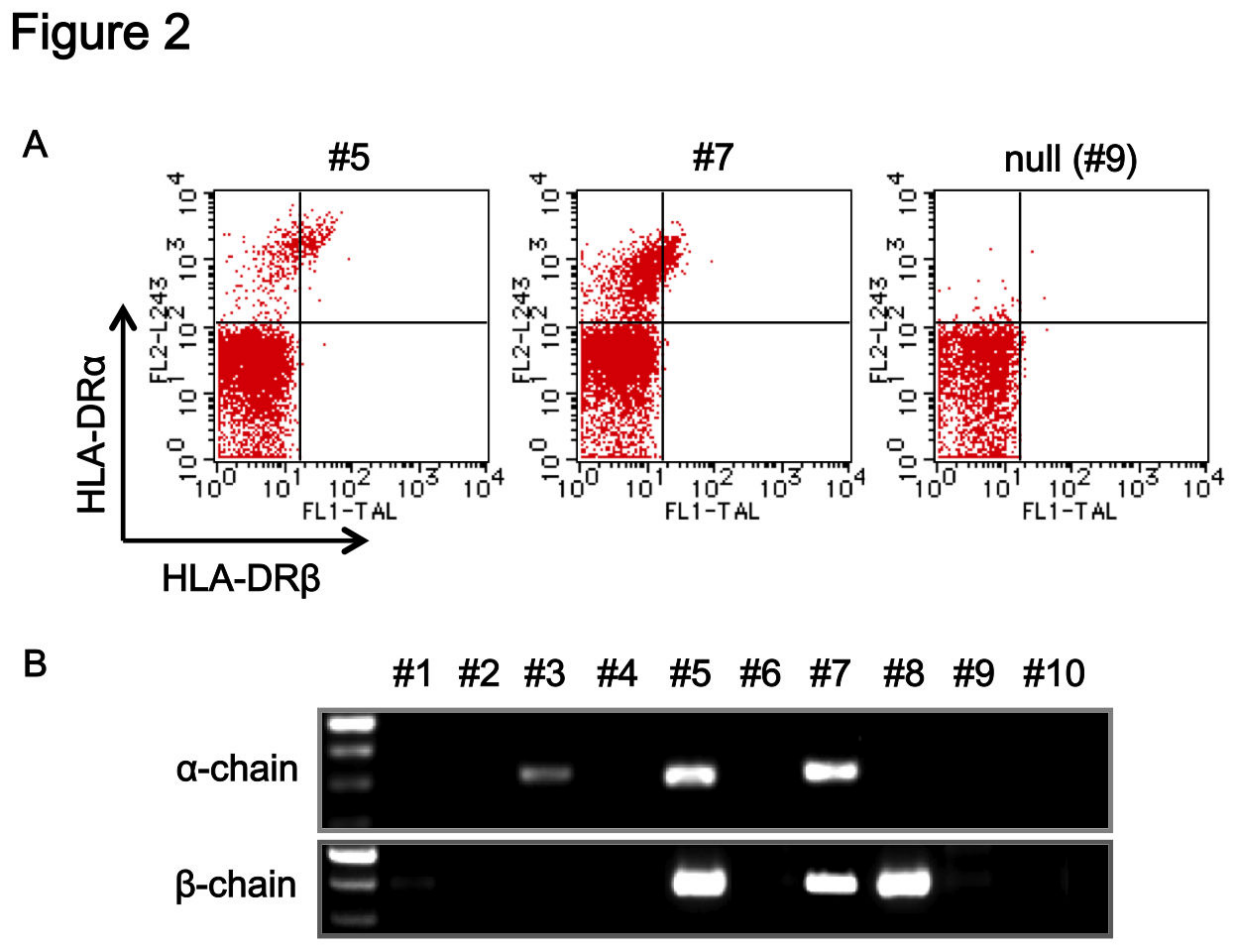
Selection of transgene-positive founder mice (F_0_). Twenty five F_0_ mice were arbitrarily numbered #1~#25. PBMCs were analyzed for HLA-DR4/I-E^d^ expression by flow-cytometric analyses (gated on lymphocytes) using anti-HLA-DRα and β mAbs (A) and genomic PCR analyses (B). A typical dot blot of PBMCs from other mice negative for transgenes is shown (null (#9) in A) and results from representative 10 mice from 25 F_0_ mice are shown in B.

**Figure 3:**
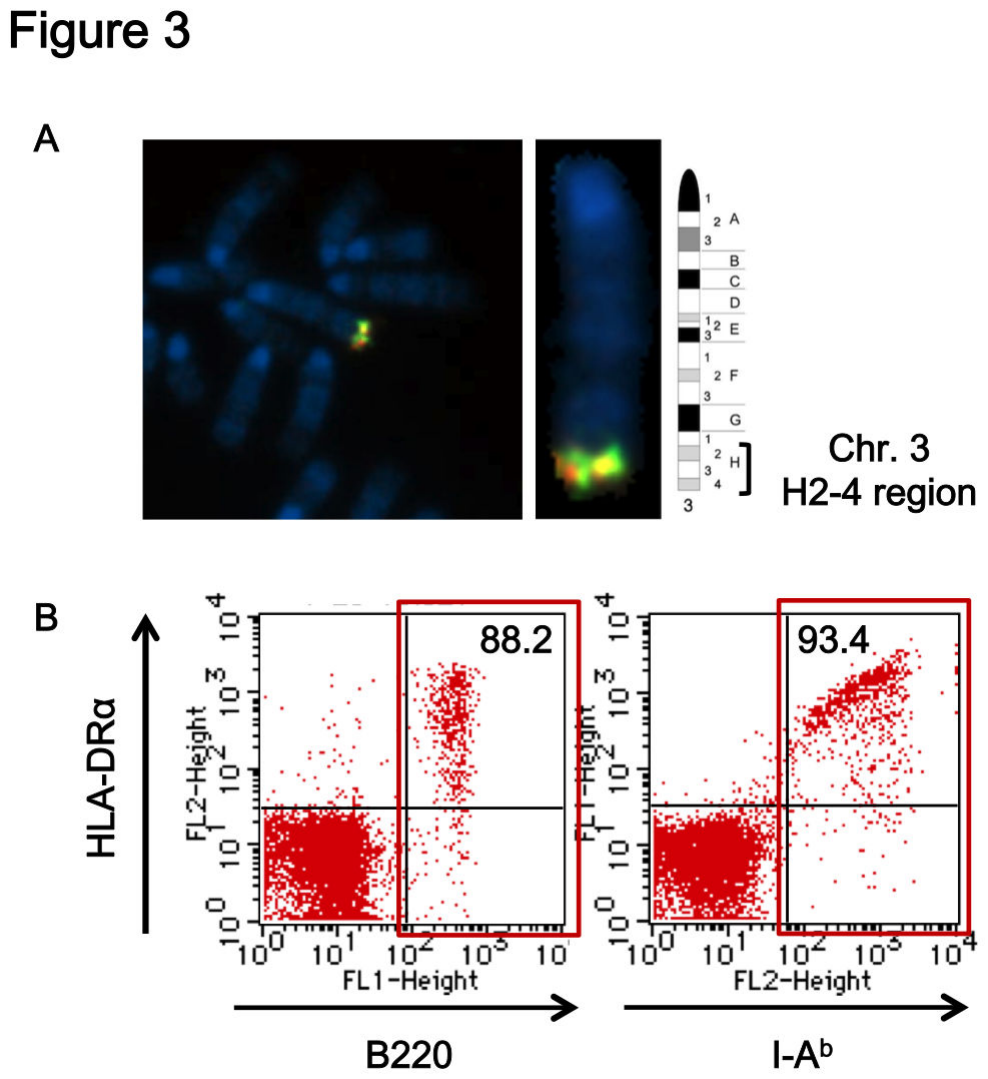
Chromosomal localization of transgene insertion site and cell-type specific expression of HLA-DR4/I-E^d^. (A) Mapping of transgene insertion by fluorescence in situ hybridization (FISH) revealed integration in chromosome 3, H2-H4 region in line #5 Tgm. (B) PBMCs from line #5 Tgm were stained with anti-HLA-DR and anti-B220 mAbs (left) or anti-HLA-DR and anti-I-A^b^ mAbs (right). Numbers indicate the percentage of HLA-DR4/I-E^d^-positive cells in B220-positive cells and MHC-II-positive cells indicated by the red boxes, respectively (gated on lymphocytes).

To select HLA-DR-positive F_0_ mice, their PBMCs were collected and analyzed by cell surface staining with anti-HLA-DR antibodies (anti-HLA-DRα mAb L243 and anti-HLA-DRβ mAb TAL15.1) by FACScan. Genomic DNA prepared from the cells was then subjected to PCR analysis 2 mice were positive for the expression of both HLA-DRα and -DRβ molecules (Figure 2A) and for the presence of *HLA-DRA/I-E* ^*d*^α and HLA-DRB1*0405/I-E ^*d*^β transgenes (Figure 2B), and they were designated as #5 and #7 founder mice, respectively. These two mice were maintained by crossing with WT C57BL/6 mice, and the presence and expression of the transgenes were stably transmitted to their descendants (data not shown). Fluorescence in situ hybridization (FISH) analyses using fluorescent-labeled transgenes as probes revealed that the transgenes were inserted in the H2-H4 region of chromosome 3 in the line #5 Tgm (Figure 3A) and in the B-region of Y-chromosome in line #7 Tgm (Figure S1A), respectively.

To examine which subsets of cells expressed HLA-DR4/I-E^d^ molecules, PBMCs isolated from Tgm were analyzed by flow cytometry using fluorescent-labeled mAbs against HLA-DRα, mouse I-A^b^ and mouse B220. The majority of B220-positive cells were positive for HLA-DRα (Figure 3B, left panel), and most of the endogenous I-A^b^-positive cells were positive for HLA-DRα (Figure 3B, right panel), indicating the expression profile of chimeric HLA-DR4/I-E^d^ was similar to the endogenous MHC-II, (I-A^b^ of C57BL/6 mice). Similar observation was also obtained in #7 Tgm (Figure S1B). We noticed that the expression level and positive ratio of HLA-DR4/I-E^d^ in I-A^b^-positive cells were always slightly higher in the chromosome 3-linked line #5 Tgm compared with the Y chromosome-linked line #7 Tgm (Figure 3B and Figure S1B). Therefore, line #5 Tgm (hereafter called Tgm unless otherwise mentioned) were mainly used in the following immunological assays.

**Figure 4:**
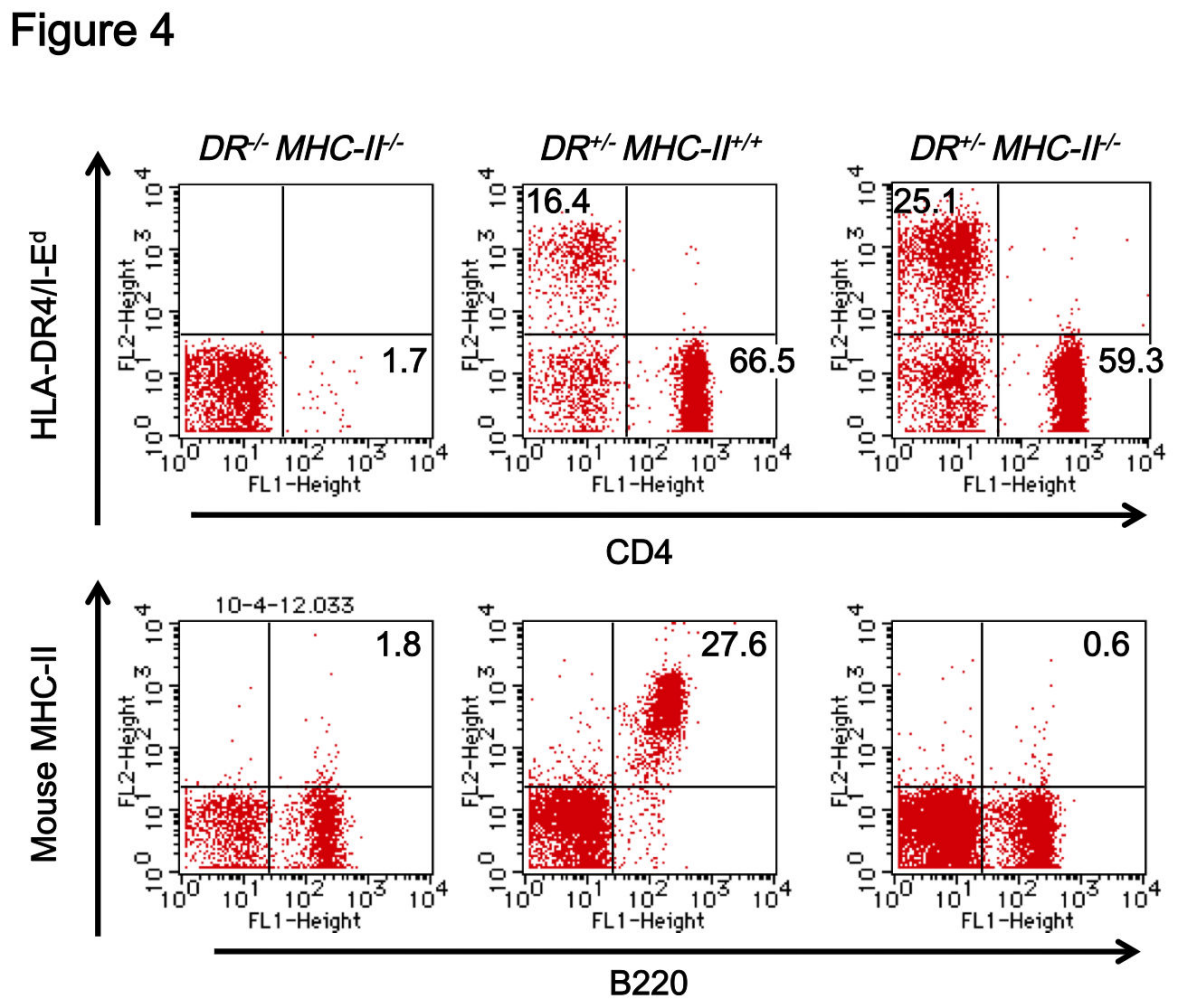
Chimeric HLA-DR4/I-E^d^ molecules induced the differentiation of mouse CD4^+^ cells. (Upper panels) PBMCs (gated on lymphocytes) from ^*MHC-II*^ knock-out mice (DR^-/-^MHC-II^-/-^), Tgm (DR^+/-^MHC-II^+/+^) and Tgm that lack ^*MHC-II*^ (DR^+/-^MHC-II^-/-^) were stained with anti-HLA-DR and anti-mouse CD4 mAbs. (Lower panels) PBMC (gated on lymphocytes) from each mouse was stained with anti-B220 mAb and anti-mouse MHC-II mAb.

To examine the function of chimeric HLA-DR4/I-E^d^ molecules, Tgm that lacked expression of endogenous ^*MHC-II*^ gene were generated by crossing Tgm with ^*MHC-II*^ knock-out mice (B6.129S2-H2^*dlAb1-Ea/J*^, Jackson Laboratory) and the presence of CD4^+^ cells in PBMCs was examined. In PBMCs of ^*MHC-II*^ knock-out mice (*DR* ^*-/-*^ *MHC-II* ^*-/-*^, Figure 4 left panels), both MHC-II positive cells (1.8%, lower panel) and CD4^+^ cells (1.7%, upper panel) were almost absent. In PBMCs of Tgm (*DR* ^*+/-*^ *MHC-II* ^*+/+*^, Figure 4 center panels), both MHC-II positive (27.6%, lower panel) and HLA-DR4/I-E^d^ positive (16.4%, upper panel) cells were present and normal amount of CD4^+^ cells were present (66.5%, upper panel). In PBMCs of *DR* ^*+/-*^ *MHC-II* ^*-/-*^ mice (Figure 4 right panels), although MHC-II positive cells were absent (0.6%, lower panel), the expression of HLA-DR4/I-E^d^ (upper panel) in the mice restored the presence of CD4^+^ cells (59.3%, upper panel). This indicated that expression of either HLA-DR/I-E^d^ or I-A^b^ in mice were indispensable for CD4^+^ cell differentiation and since a comparable proportion of CD4^+^ cells in PBMCs of *DR* ^*+/-*^ *MHC-II* ^*-/-*^ mice with that of *DR* ^*+/-MHC-II+/+*^ mice was observed (59.3% vs 66.5%, Figure 4, upper center and right panels), the chimeric HLA-DR4/I-E^d^ molecules had an equivalent function to I-A^b^ and could induce normal differentiation of CD4^+^ cells. Thus, the chimeric HLA-DR4/I-E^d^ molecules were functionally expressed in the Tgm.

### Induction of HLA-DR4-restricted and non-self peptide-specific Th cells in Tgm

**Figure 5:**
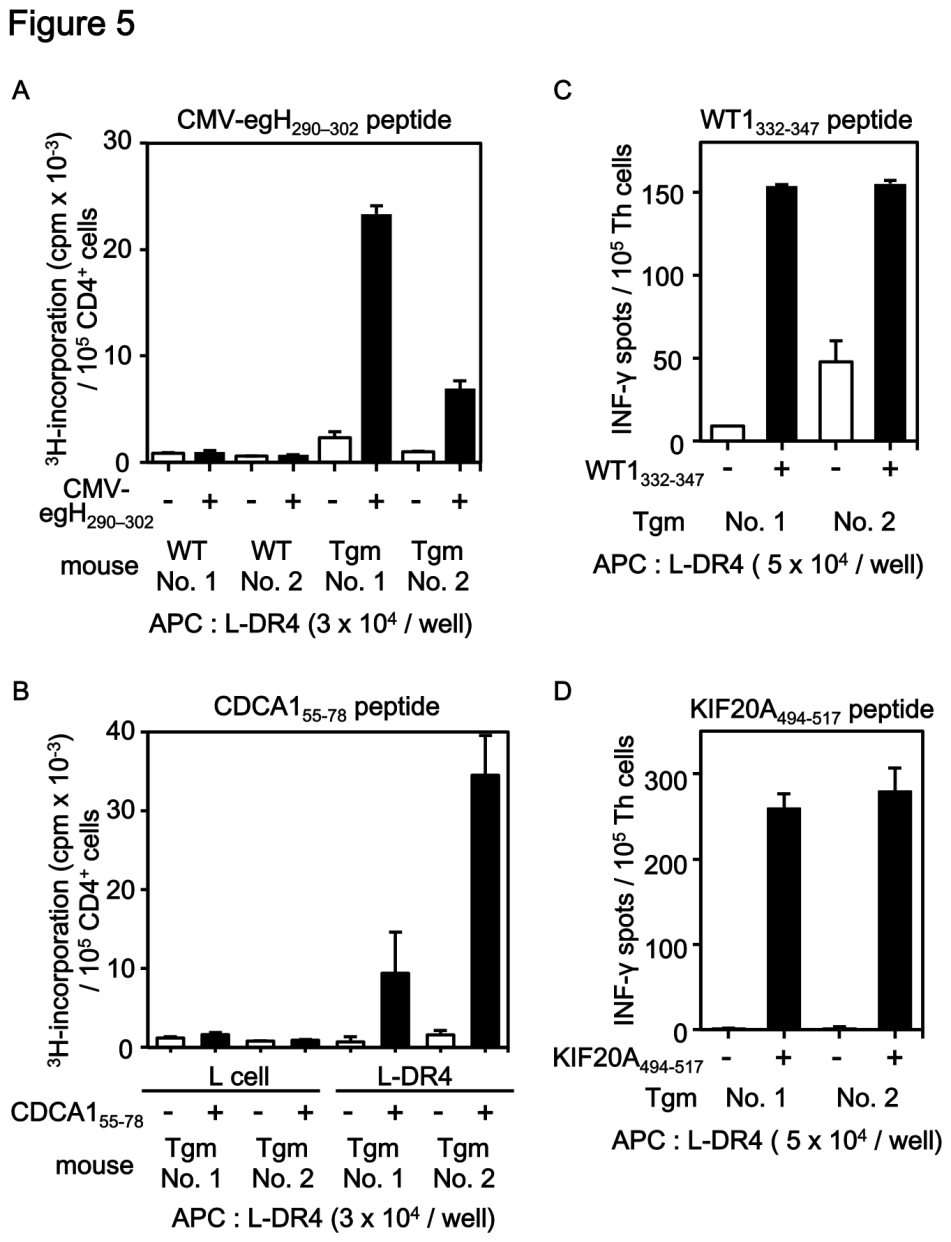
Immunization of TAA-derived peptides induced peptide-specific and HLA-DR4-restricted Th-cell responses in Tgm. Peptides emulsified in CFA and IFA were injected into the tail base of Tgm or C57BL/6 mice (WT) on day 0 and day 7. On day 14, the splenocytes were harvested and cultured *in* *vitro* for 7 days with the peptides (1 μg/ml). (A) Purified CD4^+^ cells (1 × 10^5^/well) were co-cultured with L-DR4 (3 × 10^4^/well) pulsed with or without CMV-egH_290–302_ peptide for 72 h and ^3^H-thymidine uptake was measured. (B) Purified CD4^+^ cells (1 × 10^5^/well) were co-cultured with L cells (3 × 10^4^/well) or L-DR4 (3 × 10^4^/well) pulsed with or without CDCA1_55-78_ peptide for 72 h and ^3^H-thymidine uptake was measured. (C, D) Immunization of Tgm with syngeneic BM-DCs (5 × 10^5^) pulsed with WT1_332-347_ peptide (C) or KIF20A_494-517_ peptide (D) followed by a booster shot of the peptide in CFA successfully induced IFN-γ production by Th cells (1 × 10^5^/well) in response to the peptide-pulsed L-DR4 cells (5 × 10^4^/well), but not to unpulsed L-DR4 cells.

To check that Tgm could induce peptide-specific and HLA-DR4-restricted Th-cell responses, Tgm were immunized with a CMV-derived peptide, CMV-egH_290–302_; SYLKDSDFLDAAL, that binds HLA-DR4 [12] using CFA and IFA as adjuvants. Th cells derived from immunized Tgm strongly proliferated when co-cultured with CMV-egH_290–302_ peptide-pulsed L-DR4 cells, but not with unpulsed cells (Figure 5A). In addition, splenocytes isolated from immunized WT C57BL/6 mice did not respond to either peptide-pulsed or unpulsed L-DR4 cells. Therefore, Tgm immunized with HLA-DR4-binding CMV-derived peptides successfully exhibited HLA-DR4-restricted and peptide-specific Th-cell responses. Thus, the Tgm were useful for screening of HLA-DR4-restricted Th-cell epitopes.

### Induction of Th-cell responses specific for known HLA-DR4-binding TAA-derived peptides in Tgm

Next, we determined whether immunization of Tgm with TAA-derived peptides could induce HLA-DR4-restricted peptide-specific mouse Th-cell responses. CDCA1 (cell division cycle associated 1) is a TAA frequently overexpressed in lung cancer, head-and-neck cancer and other malignancies [16,17]. CDCA1_55-78_ peptide (IVYGIRLEHFYMMPVNSEVMYPHL) is a CDCA1-derived peptide consisting of 15 amino acid residues (underlined) predicted to bind to HLA-DR4 with high affinity by a computer algorithm available at the Immune Epitope Data Base site (http://tools.immuneepitope.org/mhcii/). Tgm were immunized with CDCA1_55-78_ peptide and CFA or IFA at the tail base as described in the Materials and Methods. As shown in Figure 5B, proliferation of Th cells was observed in Th cells stimulated with peptide-pulsed L-DR4 cells, but not with unpulsed L-DR4 cells or parental L-cells pulsed with or without the peptide. The data indicated that immunization of Tgm with TAA-derived peptides predicted to bind to HLA-DR4 successfully induced HLA-DR4-restricted and peptide-specific Th cells. As we previously reported [14], the CDCA1_55-78_ peptide is naturally processed from CDCA1 protein by dendritic cells to induce HLA-DR4-restricted Th-cell responses in human PBMCs isolated from HLA-DR4-positive healthy donors and cancer patients. Thus, the screening of TAA-derived HLA-DR4 binding Th-cell epitopes could be performed by the combination of computer algorithm analyses and peptide immunization of Tgm.

The human CDCA1_55-78_ peptide is different from the mouse ortholog peptide at 3 amino acid residues (VYGIRLEHFYMMPVNSEVMYPHL vs VYGVRLEHFYMMPMNIEVTYPHL), and therefore CDCA1_55-78_ peptide is non-self and may be immunogenic in mice. However, the WT1_332-347_ peptide, a known HLA-DR4 binding human Th-cell epitope derived from Wilms' Tumor 1 antigen (WT1) [13], has an identical amino acid sequence with the mouse ortholog peptide. As expected, immunization of Tgm with WT1_332-347_ peptide using CFA and IFA as adjuvants failed to induce Th-cell responses (data not shown). DCs are superior antigen presenting cells and DC-based vaccination is thought to be a promising cancer immunotherapy [18-20]. Thus, to elicit stronger immune responses, we immunized Tgm with WT1_332-347_ peptide-pulsed bone marrow-derived (BM)-DCs with a booster shot of WT1_332-347_ peptide emulsified in CFA. In addition, to evaluate T-helper type 1 (Th1) cell-responses, which are important for the induction of potent anti-tumor immune responses [21-24], IFN-γ ELISPOT assay was performed to assess peptide-specific immune responses. As shown in Figure 5C, IFN-γ producing Th cells were increased in CD4^+^ cells stimulated with peptide-pulsed L-DR4 cells compared with those stimulated with unpulsed L-DR4 cells. Therefore, BM-DC-based peptide immunization of Tgm in combination with a th-cell assay using IFN-γ ELISPOT is an effective way to screen TAA-derived and HLA-DR4-restricted Th-cell epitopes, especially if the peptides has low immunogenicity.

Using BM-DC-based peptide immunization, we sought to identify the Th-cell epitope in KIF20A, which is frequently overexpressed in gastric cancer [25], melanoma [26], lung cancer, pancreatic cancer [27], bladder cancer, breast cancer, and various other malignancies [28], and thus is a promising target for cancer immunotherapy [29]. The 24-mer KIF20A_494-517_ peptide (TLHVAKFSAIASQLVHAPPMQLGF) consisting of overlapping 15-mer peptides with relatively high affinity binding to HLA-DR4 was predicted by a computer algorithm. In our recent study in humans, the KIF20A_494-517_ peptide induced HLA-DR4-restricted Th-cell responses from PBMCs of an HLA-DR4-positive healthy donor [15].

To investigate the immunogenicity of the KIF20A_494-517_ peptide in Tgm, mice were immunized with KIF20A_494-517_ peptide-pulsed BM-DCs, and then with KIF20A_494-517_ peptide _in_ CFA emulsion. As shown in Figure 5D, Th-cells stimulated with KIF20A_494-517_ peptide-_pulsed_ L-DR4 cells showed a large number of IFN-γ positive spots compared with the Th-cells stimulated with unpulsed L-DR4. 

### Identification of a novel TAA-derived and HLA-DR4-restricted human Th-cell epitope using Tgm

**Figure 6:**
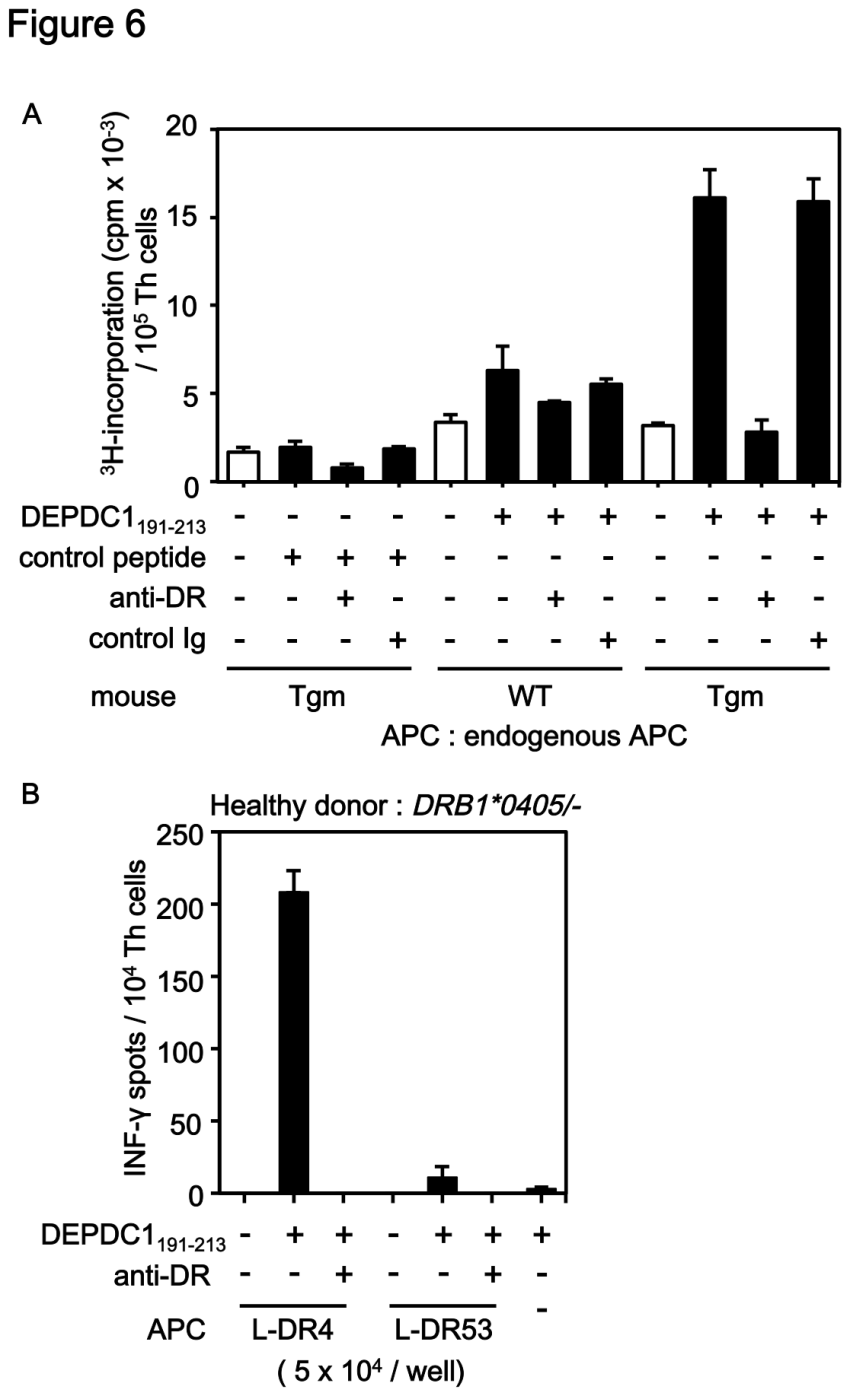
DEPDC1_191-213_ peptide induced peptide-specific and HLA-DR4-restricted Th-cell responses in both Tgm and human PBMCs. (A) Tgm and C57BL/6 mice (WT) were immunized with DEPDC1_191-213_ peptide or control peptide (DEPDC1_60-85_) in CFA and IFA as described in Figure 6 legend. On day 14, inguinal lymph node cells were harvested and *ex* *vivo* ^3^H-thymidine incorporation was measured. (B) Induced CD4^+^ T cells (1 × 10^4^/well) were co-cultured with peptide-pulsed L-DR4 (5 × 10^4^/well) or L-DR53 cells (5 × 10^4^/well) or unpulsed L-DR4 in the presence or absence of anti-HLA-DR blocking mAb L243. The CD4^+^ T cells cultured with peptide only did not produce IFN-γ.

DEP domain containing 1 (DEPDC1) is a novel TAA classified as cancer-testis antigen that is significantly overexpressed in a majority of bladder cancer specimens [30,31]. A clinical trial of DEPDC1-derived CTL epitope-vaccination was performed in six patients with advanced bladder cancers [32]. Four patients achieved stable disease or partial responses with the induction of CTL responses to DEPDC1_294-302_ 9-mer peptide, while two cases were clinically non-responsive and negative for CTL responses. To induce more effective anti-tumor immune responses, we searched several candidate Th-cell epitope peptides in DEPDC1 using the computer algorithm and selected DEPDC1_191-213_ 23-mer peptide (RYVILIYLQTILGVPSLEEVINP) consisting of overlapping 15-mer peptides with predicted high binding affinity to HLA-DR4. DEPDC1_60-85_ 26-mer peptide (NSNFGPEVTRQQTIQLLRKFLKNHVI) consisting of overlapping 15-mer peptides with predicted relatively low binding affinity to HLA-DR4 was used as a control peptide. Immunization of DEPDC1_191-213_ peptide emulsified in CFA and IFA in the tail base successfully induced the proliferation of CD4^+^ cells from immunized Tgm even by *ex vivo* stimulation with the peptide, but not from WT mice (Figure 6A). Also, immunization of Tgm with control DEPDC1_60-85_ peptide did not induce CD4^+^ cell proliferation (Figure 6A), suggesting that the peptide with predicted low binding affinity to HLA-DR4 was less immunogenic than that with higher binding affinity. The responses observed in Tgm immunized with DEPDC1_191-213_ peptide were blocked by the presence of anti-HLA-DR mAb (L243), while the presence of control immunoglobulin had no effect (Figure 6A). Thus, DEPDC1_191-213_ peptide-specific Th-cell responses were HLA-DR4/I-E^d^-restricted in Tgm.

Co-culture of CD4^+^ cells from PMBCs of an HLA-DR4-positive healthy donor with DEPDC1_191-213_ peptide-pulsed autologous CD14^+^ cell-derived DCs and PBMCs induced anti-HLA-DR mAb-sensitive Th-cell responses to the peptide-pulsed L-DR4 cells but not to HLA-DR53-expressing L cells (L-DR53, Figure 6B). Thus, Tgm in combination with the computer algorithm-based analysis demonstrated its usefulness and effectiveness for the preliminary screening of HLA-DR4-restricted Th-cell epitopes.

## Discussion

Many *HLA class II* Tgm have been established to identify Th-cell epitopes [33,34], as well as to analyze the pathogenesis of autoimmune diseases when susceptibility is associated with particular *HLA-II* alleles. In these Tgm, *HLA-II* alleles expressed in Tgm are those frequent in Caucasians but not in Japanese. To screen TAA-derived Th-cell epitopes for peptide-based cancer immunotherapy in as many Japanese as possible, we generated *HLA-DR4* Tgm expressing HLA-DRA*01:01/HLA*DRB1*04:05 genes of which allele frequency is 13.6% in the Japanese population, that is, about one forth of Japanese have this HLA-DR molecule.

Although the overall amino acid sequence homology between human and mouse CD4 is about 80%, homology of the extracellular domain is only 55% and therefore, interspecies interactions between HLA-DR4 and mouse CD4 could be a potential problem for the induction of HLA-DR4-restricted mouse Th cells. To avoid this, Tgm were generated to express chimeric HLA-DR4/I-E^d^ molecules in which only the TCR-contacting and peptide-binding α1 and β1 domains were derived from HLA-DR4 but other-domains including CD4-interacting β2 domain were derived from I-E^d^. The chimeric HLA-DR4/I-E^d^ molecules were successfully expressed on the surface of mouse L cells by co-transfection of the α and β transgenes as revealed by positive staining with anti-HLA-DR mAb, and L cell transfectants pulsed with WT1_332-347_ peptide induced HLA-DR4-restricted IFN-γ-production and WT1_332-347_ peptide-specific human Th cell clones *in vitro*. Conversely, mouse Th cells from Tgm immunized with WT1_332-347_ peptide responded to HLA-DR4-expressing L-cells in a WT1_332-347_ peptide-specific manner. Therefore, the chimeric HLA-DR4/I-E^d^ molecules were comparable with intact HLA-DR4 molecules when stimulating WT1_332-347_ peptide-specific human or mouse Th-cell responses. 

Although mice lacking endogenous I-A^b^ expression had very few, if any, CD4^+^ T cells in the periphery, HLA-DR4/I-E^d^ expression without endogenous I-A^b^ expression restored a fraction of CD4^+^ T cells in mice, comparable to mice expressing both endogenous I-A^b^ and HLA-DR4/I-E^d^ molecules. This indicated that chimeric HLA-DR4/I-E^d^ molecules were functionally equivalent to I-A^b^ molecules as they could positively select CD4^+^ thymocytes. Since the transgenes contained endogenous I-E^d^α and I-E^d^β promoter regions spanning 3.2 kb and 5.2 kb of 5'-untranslated regions, respectively, the expression of HLA-DR4/I-E^d^ was expected to be cell type- and organ-specific similar to the endogenous I-A^b^. Of total PBMCs from Tgm, more than 80% of B220-positive cells and approximately 90% of endogenous I-A^b^-positive cells were positive for HLA-DR4/I-E^d^, confirming the correct cell type-specific expression of the transgenes.

For FISH analyses, fluorescence-labeled α and β transgenes were used as probes to detect the positions of inserted transgenes. Both transgenes were co-localized in the telomeric H2-H4 region of chromosome 3 in line #5 Tgm or in the B region of the Y chromosome in line #7 Tgm with higher fluorescence intensity compared to the faint fluorescence detected on chromosome 17 where the mouse MHC region exists. This indicated that many transgenes were tandemly inserted in both Tgm lines. Nonetheless, the levels and cell-type specificity of expression of HLA-DR4/I-E^d^ and endogenous I-A^b^ were comparable between these two Tgm lines by the flow-cytometric analyses.

CMV-egH_290–302_ peptide is a cytomegalovirus envelope glycoprotein H (egH)-derived peptide reported to bind to HLA-DR4 and induced CMV-egH_290-302_ peptide-specific Th-cell responses[12]. Thus, CMV-egH_290–302_ peptide is a natural HLA-DR4 binding Th-cell epitope that could be used as a positive control to induce specific and HLA-DR4-restricted Th-cell responses in Tgm. As expected, immunization of CMV-egH_290–302_ peptide emulsified with CFA and IFA induced HLA-DR4-restricted and peptide-specific Th-cell responses. Again, mouse Th cells restricted by HLA-DR4/I-E^d^ molecules responded to CMV-egH_290–302_ peptide presented by intact HLA-DR4, indicating that HLA-DR4/I-E^d^ and HLA-DR4 were interchangeable and that Tgm could be used for screening and identification of HLA-DR4-restricted Th-cell epitope peptides.

Since the CDCA1_55-78_ peptide was predicted to be a strong HLA-DR4 binder but is a non-self peptide in mice, it was a good immunogenic peptide to induce mouse Th-cell responses in Tgm. A similar protocol to that used for CMV-egH_290–302_ peptide immunization induced CDCA1_55-78_ peptide-specific and HLA-DR4-restricted Th-cell responses. However, the WT1_332-347_ peptide has an identical amino acid sequence to the mouse ortholog peptide, and immunization with CFA and IFA adjuvants was not successful, while immunization using peptide-pulsed DC was effective for the induction of peptide-specific and HLA-DR4-restricted Th-cell responses. Therefore, to screen the computer algorithm-predicted peptides that were expected to be less immunogenic for mice, the utilization of DCs prepared from syngeneic BM-DCs could be an alternative protocol for immunization. The KIF20A_494-517_ peptide includes a 15-mer amino acid residue predicted to bind to HLA-DR4 with low affinity, and there are differences in three amino acid residues between the human peptide and mouse ortholog. However, the predicted core binding amino acid sequences to HLA-DR4 are similar between humans and mice (AKFSAIASQ vs AKFSALASQ), in which the only different amino acid residue, P6, is not in contact with the TCR but MHC-II. Thus, the KIF20A_494-517_ peptide could be less immunogenic in mice to induce apparent Th-cell responses presented by HLA-DR4/I-E^d^. However, immunization of KIF20A_494-517_ peptide_-pulsed_ BM-DCs followed by a booster shot using the same peptide in CFA emulsion successfully induced peptide-specific and HLA-DR4-restricted Th-cell responses. Therefore, the screening efficiency could be improved if peptide-pulsed DCs were used for immunization.

The DEPDC1_191-213_ peptide has a different amino acid sequence to the mouse ortholog peptide (RYVILIYLQTILGVPSLEEVINP vs RYVIMIYLQTILSLPSIEELLNP), thus immunization using CFA and IFA as adjuvants was successful. DEPDC1 is over-expressed in various cancers, especially in bladder cancer and a clinical trial using DEPDC1-derived short CTL epitope peptide-vaccination was performed for 6 patients with advanced bladder cancer [30-32], although the clinical response was marginal. In a mouse model, co-vaccination of CTL- and Th-epitope peptides from HER-2 increased the numbers of specific CTL and Th cells and decreased the number of regulatory T cells compared with CTL-epitope vaccination alone [35]. In other clinical trials, Slingluff and colleagues recently reported that combination of CTL epitope- and Th cell epitope-vaccination in melanoma patients induced clinical benefit compared with CTL epitope-vaccination alone [36], and Woods and Cebon argued that tumor-specific T-cell help was associated with improved survival in melanoma [37]. Thus, co-vaccination of the DEPDC1 CTL peptide and Th epitope identified by using Tgm may induce additional or synergistic immune responses and clinical effects for bladder cancer patients. 

In conclusion, the HLA-DR4 Tgm established in this study are useful for preliminary screening of HLA-DR4-restricted Th-cell epitope peptides derived from TAAs among candidate peptides predicted to bind to HLA-DR4 by the computer algorithm. If the Th cells from Tgm immunized with TAA peptides were reactive to syngenic BM-DCs incorporated whole TAA protein, the screened peptides could be proven to be naturally processed and such experimental system could be a powerful tool for the identification of Th-cell epitope peptides that could be applicable for vaccines for cancer immunotherapy.

## Supporting Information

Figure S1 **Chromosomal localization of transgene insertion site and Cell-type specific expression of HLA-DR4/I-E^d^ (line #7 Tgm).** (A) Mapping of transgene insertion by FISH revealed integration in chromosome Y, B-region in line #7 Tgm. (B) PBMCs from line #7 Tgm were stained with anti-HLA-DR and anti-B220 mAbs (left) or anti-HLA-DR and anti-I-A^b^ mAbs (right). Numbers indicate the percentage of HLA-DR4/I-E^d^-positive cells in B220-positive cells and MHC-II-positive cells indicated by the red boxes, respectively (gated on lymphocytes).
(TIF)
