# Supplementary figures and images for: Establishment of HLA-DR4 Transgenic Mice for the Identification of CD4+ T Cell Epitopes of Tumor-Associated Antigens
Source: PLoS One. 2013 Dec 30;8(12):e84908. doi: 10.1371/journal.pone.0084908 (PMC3875545; doi:10.1371/journal.pone.0084908)

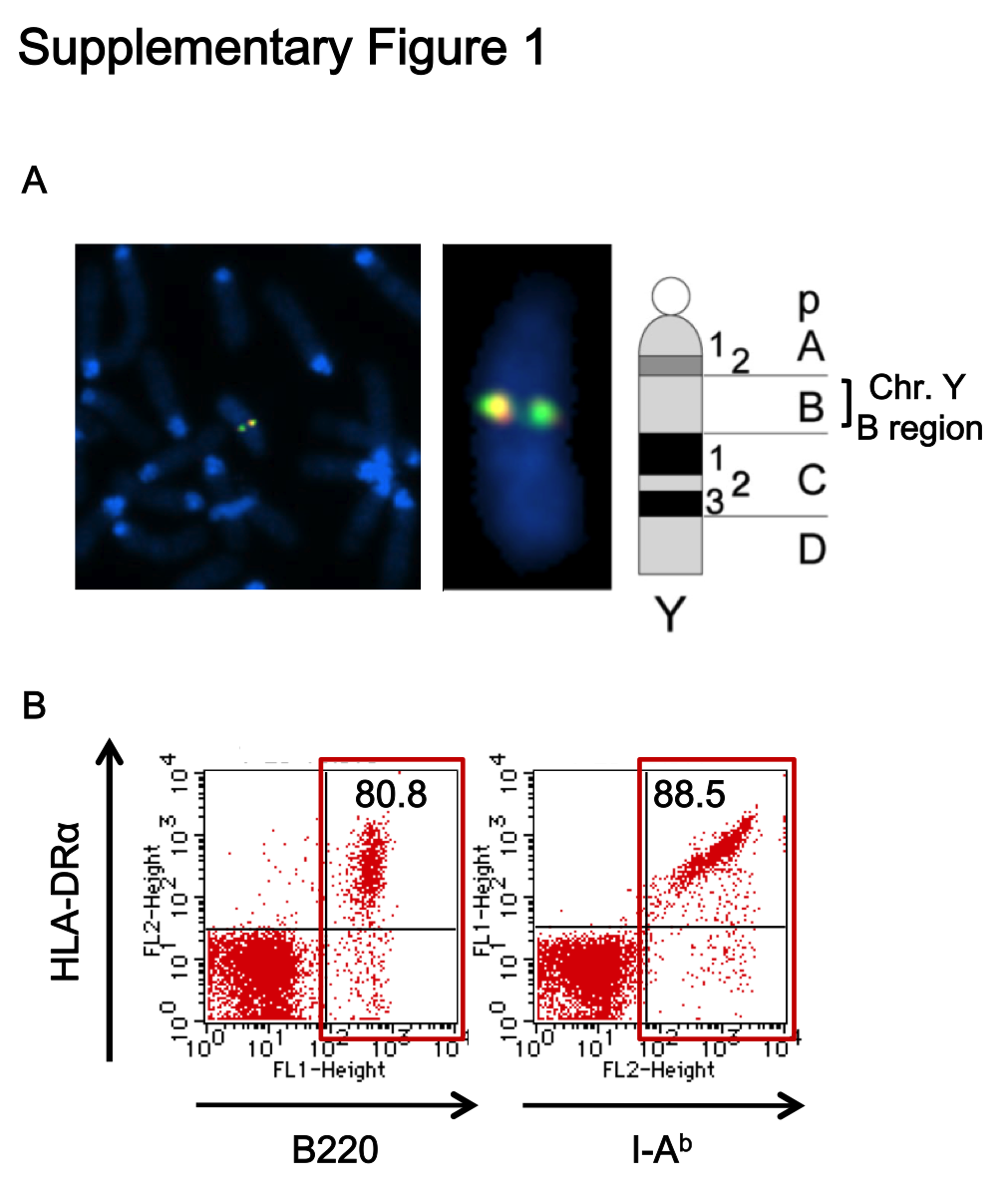

Supplement: Figure S1 — Chromosomal localization of transgene insertion site and Cell-type specific expression of HLA-DR4/I-Ed (line #7 Tgm). (A) Mapping of transgene insertion by FISH revealed integration in chromosome Y, B-region in line #7 Tgm. (B) PBMCs from line #7 Tgm were stained with anti-HLA-DR and anti-B220 mAbs (left) or anti-HLA-DR and anti-I-Ab mAbs (right). Numbers indicate the percentage of HLA-DR4/I-Ed-positive cells in B220-positive cells and MHC-II-positive cells indicated by the red boxes, respectively (gated on lymphocytes). (TIF) [file pone.0084908.s001.tif]
